# Determinants of pH-Dependent Modulation of Translocation in Dermonecrotic G-Protein-Deamidating Toxins

**DOI:** 10.3390/toxins5061167

**Published:** 2013-06-14

**Authors:** Tana L. Repella, Mengfei Ho, Brenda A. Wilson

**Affiliations:** Department of Microbiology, School of Molecular and Cell Biology, University of Illinois at Urbana-Champaign, Urbana, IL 61801, USA; E-Mails: repella@illinois.edu (T.L.R.); mho1@illinois.edu (M.H.)

**Keywords:** cytotoxic necrotizing factor, *Pasteurella multocida* toxin, dermonecrotic toxin, endosomal acidification, intoxication, drug-delivery, toxin-based therapeutics

## Abstract

Cytotoxic necrotizing factors from *E. coli* (CNF1, CNF2) and *Yersinia* (CNFy) share *N*-terminal sequence similarity with *Pasteurella multocida* toxin (PMT). This common *N*-terminal region harbors the receptor-binding and translocation domains that mediate uptake and delivery of the *C*-terminal catalytic cargo domains into the host cytosol. Subtle variations in the *N*-terminal ~500 amino acids of CNFs and PMT could allow for selective recognition of cellular receptors and thus, selective target cell specificity. Through studies with cellular inhibitors, we have identified an additional novel function for this region in modulating responses of these toxin proteins to changes in pH during intoxication and delivery of the catalytic cargo domain into the cytosol.

## 1. Introduction

Dermonecrotic toxins are members of the single-chain AB toxin family, and include the cytotoxic necrotizing factors (CNF1, CNF2, and CNF3 from *E. coli* and CNFy from *Yersinia pseudotuberculosis*), *Bordetella* sp. dermonecrotic toxin (DNT), and *Pasteurella multocida* toxin (PMT) [1,2]. Intracellular activities of dermonecrotic toxins activate G-protein-dependent, downstream mitogenic signaling pathways [3,4], which ultimately lead to activation of genes under the control of serum-response element (SRE). All dermonecrotic toxins carry a glutamine deamidase activity at their *C*-terminus that activates their G-protein target substrates and use their *N*-terminus as the machinery for binding to cells and facilitating membrane translocation of their cargo (activity domains) out of the endosomes and into the cytosol. 

CNF1 shares overall sequence homology with CNF2 (85%), CNF3 (70%) and CNFy (61%) [1]. The CNFs and DNT share sequence homology in their *C*-terminal cargo domains, which deamidate an active-site glutamine of small GTPases of the Rho family [1,3]. Although PMT does not share any *C*-terminal sequence homology with the CNFs or DNT, its *C*-terminal cargo domain also deamidates an active-site glutamine of the α subunit of heterotrimeric G proteins [2]. The CNFs and PMT also share sequence homology in their *N*-terminal domains. Receptor specificity has been shown for CNF1 and CNFy [1]. CNF1 binds to the 37-kDa laminin receptor precursor [5], which promotes internalization through the 67-kDa laminin receptor [6,7], whereas CNFy binds to heparan sulfate proteoglycan, which is also a co-receptor for CNF1 [8]. PMT binds sphingomyelin and an unidentified protein receptor [9]. It is conceivable that sequence differences in the *N*-terminus of these proteins allow for selective receptor binding and internalization. 

For many bacterial protein toxins that enter the cytosol through acidic compartments, acidification of the early endosome by a vacuolar type H^+^-ATPase (v-ATPase) is an essential step in toxin translocation and delivery of the toxic cargo into the cytosol [10]. Inhibition of acidification by reagents, such as the weak bases, methylamine or ammonium chloride, or the v-ATPase inhibitor bafilomycin A1, blocks internalization, and thereby, the cellular activity of many toxins, including CNF1 [8,11], CNF2 [12], CNFy [8], and PMT [13,14,15]. In our studies comparing the intoxication properties and cellular activities of CNF1, CNF2, and CNFy with our previous results for PMT using the SRE-luciferase reporter assay [14], we found differential responses among these toxins to endosomal acidification inhibitors. Our results, described herein, revealed additional properties in the *N*-terminal region of the toxins that could modulate the translocation efficiency of toxic cargos from acidic compartments.

## 2. Results and Discussion

### 2.1. Effect of Endosomal Acidification Inhibitors on CNF-Mediated SRE-Luciferase Activity

**Figure 1:**
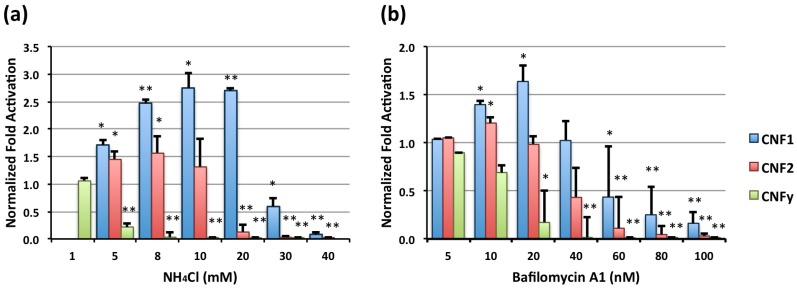
Effects of NH_4_Cl or bafilomycin A1 on cytotoxic necrotizing factor (CNF)-mediated serum response element (SRE)-luciferase activity. HEK-293T/17 cells transiently transfected with dual SRE-luciferase reporter genes were treated with the indicated concentrations of (**a**) NH_4_Cl or (**b**) bafilomycin A1 for 30 min, and then treated without or with CNF1, CNF2 or CNFy at a concentration of 100 ng/mL. After 8 h incubation cells were assayed for SRE-luciferase reporter gene activity, as described in the Experimental section. Fold activation was determined by dividing the luciferase activity measured in CNF-treated cells by the activity in untreated cells. Fold activation was then normalized to control cells that were not treated with acidification inhibitor. (*) denotes *p* value < 0.05 and (**) denotes *p* value < 0.005. In addition, Tukey’s HSD test gave *p* values of <0.0041 for CNF2-CNF1, <0.0000001 for CNFy-CNF1 and <0.0051 for CNFy-CNF2 in (**a**) and <0.000001 for CNF2-CNF1, <0.0000001 for CNFy-CNF1 and <0.00019 for CNFy-CNF2 in (**b**).

Endosomal acidification inhibitors, NH_4_Cl and bafilomycin A1, showed dose-dependent differential effects on toxin-mediated SRE-luciferase activity by CNF1, CNF2, and CNFy (Figure 1a,b, respectively). Similar to what was reported previously for PMT (IC_50_ value for NH_4_Cl ~2 mM, IC_50_ value for bafilomycin A1 ~40 nM) [14], CNFy was more sensitive to both acidification inhibitors than the other two toxins and showed dose-dependent decreases in SRE-luciferase activity with increasing inhibitor concentrations. Interestingly, CNF1, and to a lesser extent CNF2, showed an enhancement of toxin-mediated SRE-luciferase activity at lower inhibitor concentrations (maximizing around 10–20 mM NH_4_Cl or 20 nM bafilomycin A1), which was overcome at higher inhibitor concentrations. The enhanced activity observed for CNF1 was independent of incubation time for inhibitor pretreatment, as similar effects were observed with pre-incubation for 15 min as was observed for 30 min (data not shown).

**Figure 2:**
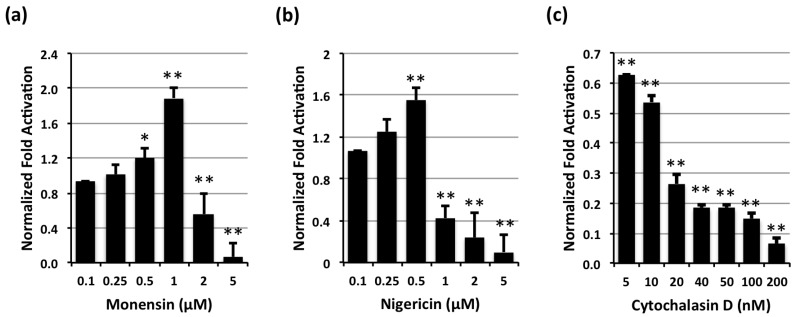
Effects of monensin, nigericin or cytochalasin D on CNF1-mediated SRE-luciferase activity. HEK-293T/17 cells transfected with SRE-luciferase reporter genes were treated without or with 100 ng/mL CNF1 and/or inhibitors at the indicated concentrations and analyzed, as described above. (*) denotes *p* value < 0.05 and (**) denotes *p* value < 0.005. (**a**) Dose effect of monensin on CNF1-mediated SRE-luciferase activity; (**b**) Dose effect of nigericin on CNF1-mediated SRE-luciferase activity; (**c**) Dose effect of cytochalasin D on CNF1-mediated SRE-luciferase activity.

Similar dose-dependent enhancement of CNF1-mediated SRE-luciferase activity was observed for other endosomal acidification inhibitors, such as monensin and nigericin (Figure 2a,b, respectively). Monensin is an ionophore that acts as a Na^+^/H^+^ antiporter [16], while the related ionophore nigericin acts as a K^+^/H^+^ antiporter. Both increase the pH of intracellular compartments and have been shown to block translocation of toxins requiring an acidic endosome step, such as diphtheria toxin [17]. However, other types of internalization inhibitors that do not affect pH, such as cytochalasin D, which blocks actin polymerization [18], did not cause the enhanced response of CNF1 (Figure 2c). These results support a model whereby some acidification of the endosome is required for translocation but moderate inhibition of the acidification process that maintains a particular pH promotes translocation of CNF1.

Potentiation of toxin activity by weak bases (nicotine, methylamine, NH_4_Cl) has been reported before for the vacuolating toxin VacA from *Helicobacter pylori* [19,20]. However, in this case it appears that the potentiation of VacA-mediated vacuolation by weak bases most likely occurred through a mechanism independent of changes in endosomal pH, since monensin inhibited VacA-induced vacuolation. Weak bases also reportedly maintain and even slightly stimulate the activity of other toxins, such as ricin, abrin, modeccin and Shiga toxin [21,22,23], but after receptor-mediated uptake into endosomes these toxins are trafficked through retrograde transport pathways to the Golgi and/or ER and translocation does not occur in acidified endosomes [23,24,25,26]. It was previously reported that in Hep-2 cells 5 mM of NH_4_Cl blocked CNF1-induced nuclear fragmentation [11], but there was no report of enhancement in CNF1-induced activity. We rationalize the discrepancy may be due to differences in experimental conditions. CNF1 is unique in that the enhanced response can be achieved with different types of acidification inhibitors, including a weak base NH_4_Cl, a proton pump inhibitor bafilomycin A, and ionophores, monensin or nigericin. These results also suggest that the source of this enhancement is related to the acid-base properties of the toxin protein itself. 

### 2.2. Effect of Nocodazole on CNF-Mediated SRE-Luciferase Activity and NH_4_Cl Enhancement of CNF1-Mediated SRE-Luciferase Activity

**Figure 3:**
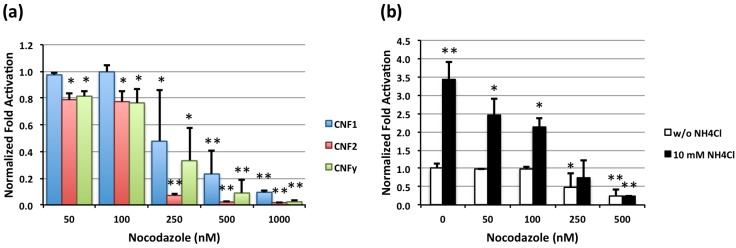
Effect of nocodazole on CNF-mediated SRE-luciferase activity and enhancement of CNF1-mediated SRE-luciferase activity by NH_4_Cl. SRE-luciferase reporter-transfected HEK-293T/17 cells were treated with the indicated toxins and inhibitors at the indicated concentrations and analyzed, similarly as described above. (*) denotes *p* value < 0.05 and (**) denotes *p* value < 0.005. (**a**) Dose effects of nocodazole on CNF-mediated SRE-luciferase activity. Tukey’s HSD test showed *p* values of <0.0000044 for CNF2-CNF1, <0.000082 for CNFy-CNF1 and <0.71 CNFy-CNF2; (**b**) Dose effect of nocodazole on the enhancement of CNF1-mediated SRE-luciferase activity by NH_4_Cl. Tukey’s HSD test showed a *p* value of <0.00005 between groups with and without NH_4_Cl.

Nocodazole, a microtubule-depolymerizing agent that disrupts microtubule dynamics and vesicle trafficking of early endosomes to late endosomes [27,28,29,30], differentially blocked toxin-mediated SRE-luciferase activity by each of the toxins in a dose-dependent manner (Figure 3a). CNF2 was more sensitive than CNF1 or CNFy, showing near complete inhibition at 250 nM nocodazole compared to >500 nM for the others; but, all three CNFs were more sensitive than PMT, which was previously shown to require concentrations > 1 µM [14]. Nocodazole also blocked the enhanced CNF1-mediated SRE-luciferase activity observed in the presence of 10 mM NH_4_Cl (Figure 3b), suggesting that the enhancement of translocation activity of CNF1 (and CNF2) occurs at the late endosome stage. However, in the absence of NH_4_Cl, the concentration required for nocodazole blockage of CNF1 activity is >100 nM, and the NH_4_Cl-induced enhancement is partially blocked at lower nocodazole concentrations. This suggests that there may be two separate pathways (or mechanisms) for CNF1 translocation defined by nocodazole action. This is further supported by the greater sensitivity to nocodazole observed for CNF2, which suggests that CNF2 may be more dependent on transport to the late endosome for translocation activity.

### 2.3. Comparison of CNF-Mediated SRE-Luciferase Activity

**Figure 4:**
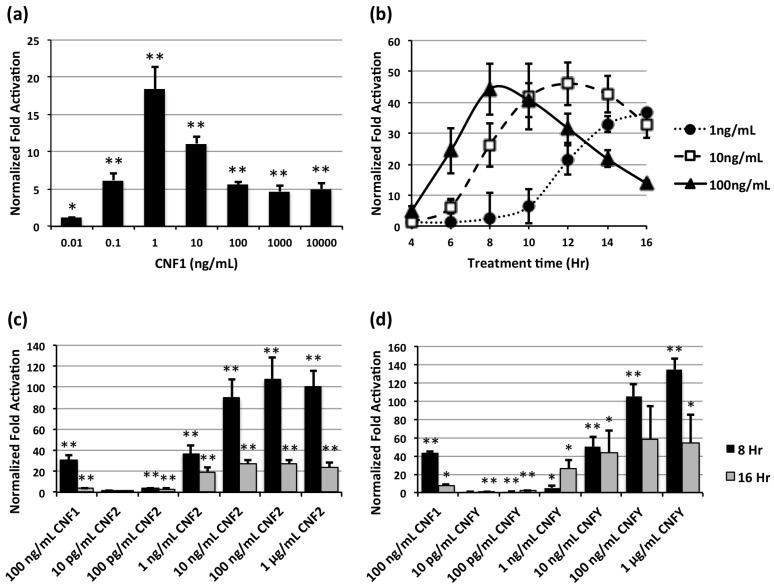
Time- and dose-dependent responses of CNF-mediated SRE-luciferase activity. SRE-luciferase reporter-transfected HEK-293T/17 cells were treated with the indicated toxins for the indicated times and concentrations and analyzed, similarly as described above. (*) denotes *p* value < 0.05 and (**) denotes *p* value < 0.005. (**a**) Dose response of CNF1-mediated SRE-luciferase activity after 16 h; (**b**) Time course of dose response of CNF1-mediated SRE-luciferase activity; (**c**) Dose response of CNF2-mediated SRE-luciferase activity after 8 or 16 h of toxin treatment, compared to CNF1; (**d**) Dose response of CNFy-mediated SRE-luciferase activity after 8 or 16 h of toxin treatment, compared to CNF1.

We considered the possibility that the observed differences in SRE-luciferase activity for each of the toxins might be attributed to differences in potency or optimal cell receptor interactions. To address this, we determined the time- and dose-dependencies of the SRE-luciferase activity for CNF1 (Figure 4a,b) and compared at two different times (8 h and 16 h) the responses to CNF1 at a fixed dose of 100 ng/mL with varying doses of CNF2 or CNFy (Figure 4c,d, respectively). For each toxin the responses were both time- and dose-dependent. We first noticed that after treatment with CNF1 for 16 h, the SRE-luciferase activity peaked at 1 ng/mL (Figure 4a). It was determined that at 100 ng/mL of CNF1, the SRE-luciferase activity peaked at 8 h, and the peak time for 10 ng/mL and 1 ng/mL was 12 and 16 h, correspondingly. In comparing the three toxins under optimal experimental conditions for CNF1, CNF2 was the most potent of the toxins, requiring 100-fold less toxin than CNF1 to achieve the equivalent fold activation of reporter activity as mediated by CNF1 at 100 ng/mL for 8 hrs exposure (Figure 4c). Similarly, CNFy was found to be 10-fold more active than CNF1 (Figure 4d).

### 2.4. Comparison of Selected *N*-Terminal Regions of CNFs and PMT

**Figure 5:**
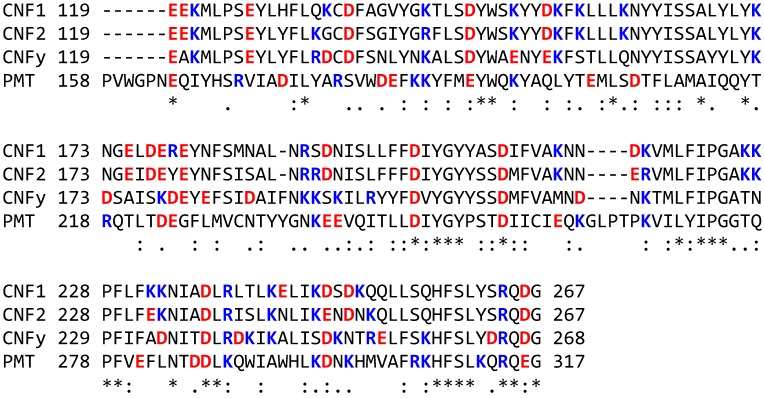
Comparison of selected *N*-terminal sequences of the CNFs and PMT. Shown in red are acidic residues (D, E), in blue are basic residues (K, R). (*) denotes identical residues, (:) denotes conserved residues with highly similar properties, (.) denotes residues with weakly similar properties.

One possible explanation for the observed potentiation of CNF1 (and CNF2) responses at lower concentrations of acidification inhibitors might be that they provide an endosomal pH environment that is more favorable for translocation during trafficking to the late endosome and then the lysosome. On the other hand, higher concentrations of these acidification inhibitors might raise the pH to a point where translocation itself is unfavorable, and thereby blocking toxin-mediated reporter gene activity. Examination of the protein sequences of the CNFs and PMT revealed differences in the net number of positively and negatively charged amino acid residues in the *N*-terminal regions of CNF1 and CNF2 compared to CNFy and PMT (Figure 5). The most drastic contrast in charges can be found between residues corresponding to 119 and 267 of CNF1. For this putative activity-modulating domain (119–267), there is a net charge of +2 for CNF1 and +1 for CNF2, while for CNFy and PMT there is a net negative charge of −4 and −3, correspondingly (Table 1). This differentially charged, putative activity-modulating domain is upstream of the proposed translocation-initiating twin hydrophobic helices (residues 330–420) [11,31]. It is conceivable that some interaction exists between the negatively charged twin helices and the positively charged activity-modulating domain (in CNF1 and CNF2). Unlike for CNFy or PMT, where both regions are negatively charged, moderate acidification (pH > 6) might be more favorable than the normal late endosomal environment (pH < 5.5) for allowing conformational changes for optimal translocation of CNF1. However, application of an external pH pulse was reported to induce direct transfer of CNF1 activity from the plasma membrane into the cytosol [11], and was shown to have a preference for pH < 5.2. The source of this discrepancy may lie in differences between the environment of membrane-bound toxin and that of endosome-localized toxin. It also suggests the possibility of multiple acidification-dependent translocation determinants for CNF1.

**Table 1:** Comparison of selected *N*-terminal regions of the CNFs and PMT.

	Calc. pI value ^a^				Residues 119–267 ^b^			
	1–536 ^b^	1–267	267–536	1–119	Number R/K	Number D/E	Net Charge	Calc. pI
CNF1	5.05	6.34	4.60	4.88	21	19	2	8.48
CNF2	5.66	6.84	5.15	5.58	20	19	1	7.97
CNFy	4.80	5.17	4.57	5.20	18	22	−4	5.00
PMT	4.99	5.34	4.70	5.18	16	19	−3	5.80

^a^ pI values were calculated using ExPASy pI/MW calculator [32]; ^b^ Numbers correspond to CNF1 amino acid residues.

## 3. Experimental Section

### 3.1. Expression, Purification and Quantification of CNF Proteins

Recombinant CNF proteins were expressed and purified as previously described for PMT [14,33]. CNF1 was expressed under the induction of IPTG in *Escherichia coli* XL1-Blue containing the plasmid vector pQE-CNF1, obtained as a generous gift from Dr. Alison O’Brien at the Uniform Services University. CNF2 was expressed under the induction of IPTG in *E. coli* XL1-Blue containing the plasmid vector pProEx-CNF2, obtained as a generous gift from Dr. Eric Oswald at the Centre de Physiopathologie de Toulouse-Purpan. CNFy was expressed under the induction of IPTG in *E. coli* BL21 containing the plasmid vector pQE-CNFy. The pQE-CNFy was generated by PCR amplification of the CNFy gene from genomic DNA isolated from *Yersinia pseudotuberculosis* strain YPIII, which was obtained as a generous gift from Dr. James Bliska at SUNY/Stonybrook, and fragment exchanged into the pQE-CNF1 plasmid vector. 

Briefly, the recombinant proteins were purified from their respective cell extracts by Ni^2+^-NTA-agarose chromatography (Qiagen, Valencia, CA, USA). Fractions containing toxin were further purified by FPLC using HiTrapQ anion exchange chromatography (Amersham-GE Healthcare Life Sciences, Pittsburgh, PA, USA) and desalted with a PD-10 column (Amersham-GE Healthcare Life Sciences, Pittsburgh, PA, USA). The toxins were further purified by FPLC using a HiTrapQ anion exchange column and a Superdex 200 sizing column (GE Healthcare Life Sciences, Pittsburgh, PA, USA). Fractions containing toxin were concentrated using Centricon filter units (Millipore, Billerica, MA, USA) and desalted using a PD-10 column (GE Healthcare Life Sciences, Pittsburgh, PA, USA) with phosphate-buffered saline (PBS) containing 10% glycerol. The concentration of toxin was determined by NIH Image J digital image analysis of SDS-PAGE gels stained with Pierce GelCode Blue (Thermo Scientific, Rockford, IL, USA), using bovine serum albumin (BSA) as the standard. Toxin samples were stored at −80 °C until use.

### 3.2. Cell Culture

HEK 293-T cells (ATCC # CRL-11268) were cultured and maintained at 37 °C and 5% CO_2_ in DMEM (Gibco-Invitrogen, Grand Island, NY, USA) with 10% fetal bovine serum (FBS, Atlanta Biologicals, Lawrenceville, GA, USA), 100 U/mL penicillin G, and 100 µg/mL streptomycin.

### 3.3. SRE-Luciferase Assay

HEK 293-T cells at 80% confluence were replated at a 1:7 ratio in 24-well plates. The next day the medium was changed to DMEM with 2% FBS, penicillin and streptomycin, and cells were transfected using the calcium phosphate method [34]. The plasmid DNA (0.25 µg/mL of pSRE-*luc* (Stratagene-Agilent Technologies, La Jolla, CA, USA) plus 0.025 µg/mL *p*-Renilla-TK (pGL 7.4 hRluc/TK, Promega, Madison, WI, USA)) in a solution of 250 mM CaCl_2_ was added dropwise to a solution of 2× HEPES-buffered saline while vortexing. The solution was incubated at room temperature for 20 min and then added dropwise to each well. Cells were incubated for 7 h, after which the indicated toxin was added at the indicated final concentration. After treatment for the indicated time, the medium was removed, and cells were lysed by adding 150 µL of 1× Passive Lysis Buffer (Promega, Madison, WI, USA) and incubating for 15 min on a rotary shaker. Cell lysates were analyzed using the Dual Luciferase Assay System (Promega, Madison, WI, USA), according to manufacturer’s protocol. Luminescence was measured using a Synergy-HT multi-detection microplate reader (BioTek, Winooski, VT, USA) and results were reported as relative light units (sensitivity = 100, integration time = 1 s).

### 3.4. Data Analysis

SRE-luciferase activity was determined by dividing the firefly luciferase activity by the *Renilla* luciferase activity. Within each experiment triplicates of SRE-luciferase activity was averaged and the average of the toxin-treated SRE-luciferase activity was divided by the average of the untreated SRE-luciferase activity to determine the fold activation. In the inhibitor experiments the fold activation for the inhibitor-treated groups was divided by the fold activation of the untreated groups to obtain the fold activation, normalized to control. Data is expressed as the mean ± S.D. of results from at least three independent experiments repeated in triplicate. Student’s *t*-test, calculated in Microsoft Excel, was then used to compare the normalized fold-activation values of the treated and untreated samples in the experiments using chemical inhibitors. Where indicated, (*) denotes *p* value < 0.05 and (**) denotes *p* value < 0.005. For some experimental series, two-way ANOVA analysis, followed by Tukey’s honestly significant difference (HSD) test was performed using R Package, aov and Tukey HSD.

### 3.5. Treatment of Cells with Toxins and Inhibitors

HEK 293-T cells at 80% confluence, maintained as described above, were plated at a 1:7 ratio in 24-well plates. The next day the medium was changed to DMEM with 2% FBS, penicillin and streptomycin, and cells were transfected, as described above. Cells were incubated for 7 h at 37 °C after which medium containing the indicated inhibitor at the indicated final concentration was added to the wells. Stock solutions of 700 nM bafilomycin A1 (Alexis Biochemicals, San Diego, CA, USA), 100 µM nocodazole (Sigma, St. Louis, MO, USA) and 200 µM cytochalasin D (Sigma, St. Louis, MO, USA) were generated by dissolving the inhibitor in dimethyl sulfoxide (DMSO). Stock solutions of 70 µM monensin (Sigma, St. Louis, MO, USA) and 35 µM nigericin (Sigma, St. Louis, MO, USA) were generated by dissolving the inhibitor in methanol. A stock solution of 350 mM NH_4_Cl (J. T. Baker-Avantor, Center Valley, PA, USA) was generated by dissolving NH_4_Cl in water. After 15 min incubation with the inhibitor, the indicated toxin was added to the wells at a final concentration of 100 ng/mL. After 8 h of toxin treatment, the medium was removed and the cells were lysed by adding 150 µL of 1× Passive Lysis Buffer (Promega, Madison, WI, USA) and incubating for 15 min on a rotary shaker. Cell lysates were analyzed using the Dual Luciferase Assay System (Promega, Madison, WI, USA), according to the manufacturer’s protocol. Luminescence was measured using a Synergy-HT multi-detection microplate reader (BioTek, Winooski, VT, USA). SRE-luciferase activity was determined as described above, and the data were expressed as the mean ± S.D. of results from three independent experiments repeated in triplicate.

## 4. Conclusions

We report here differential toxin responses to acidification inhibitors among three closely related toxins. Namely, we found that moderate concentrations of inhibitor enhanced the activity of CNF1 and to a lesser extent CNF2, whereas this potentiation of activity was not observed for CNFy or for PMT, as previously reported [14]. Notably, this potentiation of toxin activity was pH-dependent, as similar responses were observed for acidification inhibitors that work through different mechanisms: a weak base NH_4_Cl, a v-ATPase pump inhibitor bafilomycin A1, and two ionophores monensin and nigericin. This suggests that the observed differential responses could be attributed to differences in the acid-base properties of the toxin proteins. Sequence comparison among the toxins identified a putative activity-modulating domain, corresponding to CNF amino acids residues 119–267, that might be responsible for the observed potentiation. Further structure-activity studies are required to verify the mechanism for the observed properties of this activity-modulating domain.

Our findings point to the possibility that modulation of protein translocation properties could be achieved through structural determinants that alter acidification responses and enhance translocation efficiency. This paradigm has potential application in designing improved protein-based therapeutic delivery systems, such as immunotoxins [35,36,37] and other toxin-based strategies [38,39]. 
